# The impact of technological innovation on renewable energy production: accounting for the roles of economic and environmental factors using a method of moments quantile regression

**DOI:** 10.1016/j.heliyon.2022.e09913

**Published:** 2022-07-09

**Authors:** Sakiru Adebola Solarin, Mufutau Opeyemi Bello, Aviral Kumar Tiwari

**Affiliations:** a School of Economics, University of Nottingham - Malaysia Campus Jalan Broga, 43500, Semenyih, Malaysia; b Faculty of Social Sciences, Department of Economics, University of Ilorin, PMB 1515, Ilorin, Nigeria; c Indian Institute of Management Bodh Gaya, Uruvela, Prabandh Vihar, Bodh Gaya, 824234, Gaya, Bihar, India

**Keywords:** Renewable energy innovation, Renewable energy production, Sustainable development, BRICS, Panel quantile regression

## Abstract

Advancement in renewables is one of the most effective techniques for sustained long-term development, and nations across the globe are making efforts to change their economic and industrial structures in a bid to boost green growth. With the advent of the Fourth Industrial Revolution (4IR), the availability, access, and use of green technologies including renewable energy have significantly improved. Researches on the factors that influence renewable energy production are available. However, we are unaware of any previous research that examines the role of renewable energy innovation in the promotion of renewable energy production. As a result, this study evaluates the impact of technical innovation on green growth from 1993 to 2018, while accounting for real GDP, producer price index, and CO_2_ emissions. Due to their pivotal status among the developing countries, our study has focused on the BRICS countries. By using a new panel quantile regression augmented with the method of moments, the empirical findings suggest that the influence of renewable energy innovation on renewable energy production is significantly positive across all quantiles. Moreover, the coefficients are generally bigger at the small quantiles, which suggests that countries with smaller renewable energy production per capita (India and South Africa) have a higher probability to experience a greater impact of renewable energy innovation per capita than countries with bigger renewable energy production per capita (Brazil and Russia).

## 1 Introduction

The continuous rise in global warming with its attendant negative effects on the climate has heightened the need to decarbonize the global energy sector. The most important, fastest, and cheapest step to achieve this decarbonization agenda is the development of energy from renewable sources (Rogelj et al., 2013)**.** Essential to the successful actualization of the decarbonization agenda, especially in this age of industrial revolution 4.0, is the extent of innovation in energy resources. Consequently, nations across the globe are actively ramping up their energy innovation endeavors in order to decarbonize the global energy grid and achieve the net-zero carbon goals. This has been aided by the new wave of industrial revolution 4.0 as investors and manufacturers have already keyed into this trend by turning their attention to energy-related technological innovations that can help design smart energy grids for today and future needs. This development has, among others, already resulted in the implementation of a number of technological innovations that can enhance renewable energy resources such as the deployment of artificial intelligence-powered robots to monitor the efficiency of hydropower plants and lower the cost of maintenance, the development of hydropower battery hybrids to improve grid services, and the adoption of a ‘hyperloop for fish’ to safely transport fish past dams (International Hydropower Association, 2021).

Notwithstanding the emergence of the fourth industrial revolution (4IR), the advancement of 4.0, the development of these innovations requires substantial investment and as such, there is a need to assess their effectiveness in terms of actually helping to achieve the decarbonization agenda through the promotion of cleaner, renewable energy (Modis, 2019; Sharifi et al., 2019). Despite the need to take stock of the true end-result of technological innovations’ impact on the advancement of renewable energy resources, empirical exercises in this area have been relatively passive, as most research efforts have focused mostly on the development of these innovations without measuring their effects on the promotion of energy from clean resources. We, therefore, contribute to this strand of research by focusing on the BRICS countries, and this is important due to several reasons.

First, nations within the BRICS are leading the way in industrial revolution 4.0 as it relates to innovations in renewable energy^1^. For instance, having already taken the credit for building the world’s largest hydropower station known as the Three Georges Dam with a potential to generate 22.5 GW (GW) of electricity and a project cost of about US$29 billion as far back as 1994, China is poised to complete the construction of Baihetan hydropower station, which is a 16 GW hydroelectric facility by July 2022. The facility is estimated to cost around 220 billion yuan ($34.07 billion) upon completion (NS Energy, 2021). Other energy innovation solutions in China include the use of Artificial Intelligence (AI) to monitor power plants and the development of the Hydropower Smart Remote O&M System from 29-patented inventions and four proprietary software solutions. Operators in China have also developed AI innovative tools to aid automation and optimize inspection and maintenance at their power plants (International Hydropower Association, 2021). India aims to have 175 GW of energy from renewables by 2022, with solar energy, wind power, biopower, and small hydropower projects accounting for 100 GW, 60 GW, 10 GW, and 5 GW, respectively (Paulraj et al., 2019). Interestingly, private investors, both foreign and local have pledged to attain a total capacity of over 270 GW, which is far beyond the lofty target of 175 GW. The pledges include 58 GW from foreign entities and 191 GW from private businesses. The rest is made up of 18 GW from the private sector while Indian Railways is responsible for 5 GW (National Institution for Transforming India, 2017). According to recent predictions, solar capacity will exceed 750 GW and wind capacity will exceed 410 GW by 2047 (India Smart Grid Forum (ISGF), n.d.; Sholapurkar & Mahajan, 2015).

The Energy Big Push (EBP) agenda was launched in Brazil in a bid to hasten a carbon-neutral and sustainable energy transition. The Big Push for Sustainability is a set of policies that use local and foreign investments to create a worthy cycle of economic expansion, job creation, income distribution, elimination of inequalities and structural gaps, and environmental sustainability (Economic Commission for Latin America and the Caribbean (ECLAC), 2020). EBP agenda requires Brazil to support a large push for investments in the country, with an emphasis on innovation in energy from clean sources. In 2015, Brazil invested about US$206 million in energy innovation, with renewable energy accounting for around 47% of the total (Economic Commission for Latin America and the Caribbean (ECLAC), 2020). In Russia, Ecoflot, a solar energy-powered autonomous floating platform that provides topical aeration, was implemented in 2020 (International Hydropower Association, 2021). Russia is also increasing its energy efficiency and modernizing its energy system through decarbonization, energy-saving, and the depolyment of renewable energy (Strielkowski et al., 2021). According to Chebotareva et al. (2020), renewable energy technologies are also driving the development of economically feasible regional energy development programs in Russia. This has been propelled by the requirement to promote advanced energy technologies, increase the energy efficiency of production and comply with the environmental laws (Chebotareva et al., 2020).

Furthermore, patents for renewable energy innovations in the BRICS countries have increased significantly within the last three decades. For instance, in China, the number of renewable energy patents increased from 79 in 1993 to 18,027 in 2018 while that of Russia increased from 109 to 186 within the same period. The figure went up from 32 to 240 in Brazil, and from 14 to 59 in India while South Africa’s figure went up from 10 to 93 between 1993 and 2018. Overall, the BRICS countries recorded a combined renewable energy patent of 142,335 between 1993 and 2018 ((OECD), 2021). Renewable Energy Independent Power Producer Procurement Programme (REI4P) was started in South Africa to reduce CO2 emissions by about 32% by 2030 through the installation of 17.3 GW of small renewable projects including biomass, wind, solar, wind, and biogas, as part of its Integrated Resource Plan (Walwyn and Brent, 2015). Recognizing that innovation adds vital impetus to the fundamental economic change required for economic expansion, creation of employment opportunities, and improved standard of living, the South African authorities sought to increase the nation's research and development investment by 100 percent (Department of Energy, 2017).

Moreover, with a combined gross domestic product (GDP) of US$20.58 trillion (at constant 2010 US$) the BRICS countries alone accounted for almost a quarter (24.3%) of the global GDP in 2020 (The World Bank, 2021). On the basis of the amount of energy consumed, the BRICS countries also have a massive footprint as they consumed 222.64 EJ, an equivalent to 40% of the global primary energy consumption in 2020 (British Petroleum, 2021). With about 11.4 EJ representing 36% of the global energy consumption from renewables, the BRICS nations are the biggest global consumers surpassing both Europe (8.94 EJ) and North America (7.04 EJ) in second and third positions respectively in 2020 (British Petroleum, 2021). With a human population of more than three billion, the BRICS nations constitute over 41% of the entire population of the world (The World Bank, 2021). In addition to their population endowment, which reflects in low labour costs, the BRICS nations are also endowed with technological innovations as well as mineral resources (Zaman et al., 2016).

Despite the emergence of industrial revolution 4.0 and the considerable efforts made to advance the development of renewable energy innovations and tremendous potential for renewable energy within the BRICS countries, the countries are still faced with serious environmental and climate change challenges. For instance, about 31983.6 million tonnes of carbon dioxide, representing 45% of global carbon emission, was emitted by the BRICS counties in 2020 (British Petroleum, 2021). According to the Global Footprint Network, three out of the five countries that make up the BRICS have deficit bio-capacity (the amount by which a country’s ecological footprint exceeds its bio-capacity) with China (302%), South Africa (207%), India (177%). Only Russia (27%), and Brazil (277%) have a positive bio-capacity reserve. With the positive bio-capacity reserve of Brazil and Russia, the BRICS countries’ bio-capacity deficit is 382%. The BRICS countries also have an average of 2.038 ecological footprints relative to the number of earths, implying that if everyone in the world lived like a resident of BRICS countries; humanity would need 1.7 earths to sustain our demand on nature. This is 0.308 higher than the global average of 1.73 (Global Footprint Network, 2021).

We add to the extant body of knowledge as follows. Firstly, this study uses innovation in renewable energy generation per capita as a proxy for technological factors. This is a departure from the previous studies that used other proxies to measure technological factors in the models involving renewable energy production including the ratio of exports of high technology to the exports of manufacturing outputs (Bamati and Raoofi, 2020) or expenditure on development and research (Przychodzen and Przychodzen, 2020). Innovations in per capita energy generated from renewables are more specific to the renewable energy sector and such proxy is a true reflection of the innovations in the renewable energy market unlike the share of technology exports which might not be connected with the renewable energy market. Spending on research and development activities might not necessarily lead to innovations if the project or research is not successful. Secondly, we have used a technique of moments quantile regression to analyze the models. This technique assists in generating the complete impacts of renewable energy innovation across the whole distribution of renewable energy production. This is because the influence of renewable energy innovation on renewable energy production can be estimated differently for nations with various levels of energy produced from renewables. Additionally, this method is robust to skewness, heteroskedasticity, and other outliers (Machado and Silva, 2019). Thus, our study might provide meaningful insights for relevant stakeholders including academic researchers, government, and policymakers involved with policies aiming to advance technological innovations in renewable energy to promote efficiency in the energy sector and also compliments existing related studies focussing on technological innovations, renewable energy, and the stabilization of the environment including Cheng et al. (2019) on BRIICS, Dong et al. (2020); Dong et al. (2021) on China, Cheng et al. (2021) on and Strielkowski et al. (2021) on Russia, among many others.

The other segments of this paper have been sectioned as follows: the review of literature is contained in the next section while the third section deals with the methodology. The penultimate section discusses and analyses the findings while the concluding section of the paper focuses on policy implications and recommendations.

### 2 Literature review

The critical impact of advancement in technology on economic growth has long been recognized within the mainstream economic growth models. Beginning with the neoclassical exogenous growth model of Solow (1956) and Swan (1956), factor productivity, captured with technological innovation, in combination with capital and labor has always been regarded as the most critical factor of economic growth. This model was augmented with the inclusion of human capital by the endogenous growth theories of Mankiw et al. (1992), Romer (1986), (1992), (1994), Lucas Jr (1988), and Rebelo (1991). Mainstream economic growth models pay little emphasis on the role of energy in the economic growth process (Ayres et al., 2013) until energy economists began to provide empirical evidence that supports the critical role of energy in the growth process (Kraft and Kraft, 1978; Proops, 1984).

As energy gains a prominent reputation as an important growth factor, research efforts on modeling the impact of innovation on energy consumption become intensified. The main interest was to determine whether innovations improve the efficiency of energy consumption. Some of the early contributors in this strand of literature include the work by Popp (2002) who analyzed data on patents in the U.S. for the period between 1970 and 1994 to assess the impact of prices of energy on innovations that are considered to be energy-efficient. Demand-side factors, which stimulate innovative activity through the increase in the value of innovations, and supply-side factors including scientific breakthroughs that enable innovations were considered. The study concludes that the quality of existing knowledge, as well as energy prices, have strong positive impacts on innovation. Similarly, Fri (2003) examined the role that knowledge acquisition plays in advancing technological innovation to enhance the energy system, while Herring and Roy (2007) assessed the rebound effect of technological innovation on energy efficiency.

Since then to date, several authors have continued to contribute to this strand of research. For instance, Jin et al. (2018) used a conventional panel data method and data from China to study the nexus between energy use and innovation and found a positive and bilateral connection between energy use and technological innovation. Their finding implies that technological innovations could assist developing countries to attain sustainability by enhancing energy efficiency and standardizing their energy structures. On their part, Pan et al. (2019a) studied the interlink between innovation, energy efficiency, and environmental regulation in China from 2006 to 2015. According to the findings, market-based environmental legislation fosters energy efficiency through technological innovation. In both the short and long term, innovation was found to play a significant influence in boosting energy efficiency.

Murad et al. (2019) used a multivariate time-series framework to look at the dynamic links between innovation, energy, and economic expansion in Denmark from 1970 to 2012. The study discovered that economic expansion has a positive effect on the use of energy, whereas energy pricing and technical innovation hurt the use of energy. The findings report both technology innovation and energy prices are determined to be a cause of energy use. As a result, the report recommends that Denmark pursue a technologically conservative energy policy. To explore the determinants of energy intensity in Indonesia, Saudi et al. (2019) used three metrics of technical innovations: investment in R&D, patents by residents, and exports that are high-tech based. The study found that energy inefficiency in Indonesia is significantly as well as negatively influenced by the three indices of technology innovation. Pan, Uddin, et al. (2019b) used yearly data from 1976 to 2014 in Bangladesh to explore the determinants of energy intensity. Technological innovation is found to influence energy intensity. Wang et al. (2021) assessed the influence of technological innovation on green total factor productivity in OECD countries. The findings reveal that innovations in technology have a strong beneficial effect on total factor productivity. According to Chen et al. (2021), in spite of the rising interest in Industry 4.0, the level of knowledge about the extent to which leakages in an economic system affect nexus between energy efficiency and innovation is still low. In this regard, the authors investigate the issue within the context of the Arab countries from 1990 to 2016. The findings show that innovations in technology have a favorable effect on the efficiency of energy, while energy efficiency is adversely affected by the development of the shadow economy.

As the world transits from carbon-intensive non-renewable energy use to carbon-neutral renewable energy to achieve green growth and sustainable energy consumption, research efforts have shifted focus from the general outlook of assessing the effect of technological innovations on the aggregate energy consumption to the more disaggregated and specific impact of technological innovation on renewable energy consumption. This is driven by the idea that if renewable energy is the future of energy consumption then the more relevant research question is whether or not innovation can enhance the consumption of renewable energy. In this regard, Geng and Ji (2016) examined how technological innovation influenced the development of renewable energy in six key advanced economies from 1980 to 2010. The findings show that there is a long-run two-way causal relationship between technical innovation and renewable energy use. This demonstrates that the promising impact of technology innovation on renewable energy production takes some time to appear.

Several authors have addressed the issue from different perspectives and incorporated several dynamics into the analysis. For instance, Alvarez-Herranz et al. (2017) studied the relationship between innovation and renewable energy use within the context of correction of air pollution levels for OECD countries, while Saudi (2019) investigated the role of non-renewable, renewable energy consumption and technological innovation within the environmental Kuznets curve (EKC) context for Malaysia and Usman et al. (2020) assessed the role of renewable energy technology as an essential determinant of fossil fuel-attributable environmental degradation in the United States. Bamati and Raoofi (2020) used a generalized least square approach to show that technological factors promote renewable energy production in 25 countries. Przychodzen and Przychodzen (2020) focused on the main political and economic factors influencing transitions to a less-carbon economy on the basis of renewable energy generation in 27 post-socialist transition countries. Increased CO_2_ emissions and per capita and higher economic growth was found to be a stimulator of renewable energy generation. The cost of production has a negative influence on energy produced from renewable sources.

Exploration of the key drivers of renewable energy consumption in OECD was carried out by Li et al. (2020) and eco-innovation innovation, income, and human capital was found to be the main drivers. Su et al. (2021) analyzed the political risk in the roles of eco-innovation and fiscal decentralization in promoting clean energy use using seven OECD countries as the reference point. The study found that eco-innovation, fiscal decentralization, and political risk are the main stimulants of renewable energy consumption. Ali et al. (2021) investigated the role of eco-innovation and renewable energy use on international trade and environmental performance in top-emitting nations.

With specific reference to the BRICS countries, some authors have also analyzed several aspects of renewable energy consumption. Dudin et al. (2016) x-rayed the problems and perspectives of transiting green economy and low-carbon energy industry in BRICS countries while Zaman et al. (2016) found that green growth is vital to sustainability in BRICS countries. Zeng et al. (2017) conducted an analysis of investments in energy from renewable sources in the BRICS countries viz-a-viz historical perspectives, models, problems, and solutions while Simonova (2019) examine structural differences in energy consumption and production, and a renewable energy development outlook among the countries that constitute the BRICS. Danish and Ulucak (2020) investigated the effect of environmental technology on green growth by regulating renewable and nonrenewable energy consumption and discovered that environmental-associated technologies facilitate green growth. The study also reveals that renewable energy encourages green growth whereas non-renewable energy hinders it. As a result, it was advised that BRICS countries should strengthen their energy sector innovations in order to achieve green growth and sustainability goals. Khattak et al. (2020) explored the effect of innovation, renewable energy use, and income on CO_2_ emissions within the framework of the EKC hypothesis. While validating the EKC hypothesis in the BRICS countries, the study established that, except for South Africa, renewable energy consumption has lessened carbon emission in the BRICS and there is a two-way causal dynamics between renewable energy usage and technological innovation.

The above x-ray of the literature reveals an important implication within the context of the current study. Most of the previous studies have focussed on investigating the impacts of renewable energy and technological innovation on improving energy efficiency and promoting sustainable development through the stabilization of the environment, these studies have largely ignored investigating the actual impact of technological innovations on the development of renewable energy generation itself. This study, therefore, adds to the extant literature by investigating the impact of technological innovations on renewable energy generation focussing on BRICS which is an important economic block leading the investment innovations around renewable energy.

## 3 Methodology

### 3.1 Modelling strategy

Modeling renewable energy production requires a multivariate framework (as against a bivariate one) as there are several possible determinants of energy production. Therefore, in addition to the main independent series-renewable energy innovation, there is a need to accommodate other relevant variables to the renewable energy production function. It is also appropriate to add more relevant determinants into a framework in order to circumvent the omission of variable bias. It is well known that omission of variable bias leads to the invalidation of the hypothesis testing and estimation. Consistent with the papers of Bamati and Raoofi (2020) and Przychodzen and Przychodzen (2020) which recognize that renewable energy is affected by technical, economic, and environmental factors, we use the following model in our empirical analysis:

(1) $$REP_{it}=\alpha_{1}REI_{it}+\alpha_{2}GDP_{it}+\alpha_{3}PPI_{it}+\alpha_{4}COE_{it}+\mu_{it},$$

where REP is the renewable energy production per capita, REI is the innovation in renewable energy generation per capita, GDP is the per capita real gross domestic product (in purchasing power parity), PPP is the producer price index and COE is the CO_2_ emissions per capita. Moreover, i (i = 1, 2 … 5) represents each country or panel member across the time frame of t (t = 1, 2 … 26). μ is the residual and is assumed to meet the classical criteria of independent as well as identically distributed with a zero mean as well as constant variance. Innovations in renewable energy generation per capita serve as the technological factor affecting renewable energy production in the framework. This is because renewable energy innovation can stimulate human capital and knowledge development, which might eventually lead to more renewable production (Przychodzen and Przychodzen, 2020). The introduction of innovative technologies improves market access and standards. Moreover, innovations can assist in the unlocking of potential power supply for underserved communities and zones through the speedy development of micro-grids based on wind, solar, or other renewable technologies. State-of-the-art solutions facilitate the integration of a greater portion of renewable energy into power systems. The integration of higher shares of renewable energy requires innovations in the entire aspects of the energy system such as out-of-the-box business models, new market design, novel system operations, as well as the enabling atmosphere, Therefore, it is anticipated that expansion in renewable energy innovation will boost renewable energy production, which is a feature of the COP26 agreement.

GDP represents one of the two economic factors affecting renewable energy production in this study. Growth in GDP implies that the income level in the economy is rising which increases the capacity of both public and private sectors to finance several projects. Rising income is necessary as renewable energy projects are usually expensive and, in most cases, costlier than those of fossil fuels. Countries experiencing growth in GDP have better prospects to access (or the capacity to develop) new technologies that are vital to the increase in the generation and usage of renewable energy (Bamati and Raoofi, 2020). Therefore, it is envisaged that expansion in GDP per capita will increase renewable energy generation.

The producer price index captures the impact of the cost of production on renewable energy generation. The rising general cost of production in the economy will negatively affect the capacity to produce renewable energy because there are several costs associated with renewable energy production. Production of renewable requires initial capital costs including capacity installation costs. Operating costs cover operations, wages, handling any waste, maintenance, and, where appropriate, costs for fuels. Other running costs include maintenance costs and repair costs. Besides, there are also the costs of switching from carbon-intensive fossil fuels to carbon-neutral renewable energy. As renewables become a bigger part power supply, additional costs are incurred, which can be due to energy storage or backup generation. Therefore, it is expected that expansion in the producer price index will decrease renewable energy generation.

Part of the main reasons for the recent popularity of renewable energy sources is climate change and global warming being experienced in several areas of the world. It is believed that greenhouse gases, especially CO_2_ emissions are key factors of climate change and global warming. Increasing CO_2_ tends to increase environmental concerns, magnify political pressure to advance green energy, and therefore generate a call for a green environment and boost the use of renewables (Omri and Nguyen, 2014). In several countries, the emission of CO_2_ is primarily to due fossil fuels' domination of the energy mix higher. Therefore, it is expected that an expansion in CO_2_ emissions will increase renewable energy production.

### 3.2 Data description and sources

Renewable energy production per capita is the total generation of hydroelectric and non-hydroelectric renewable electricity (in tonnes of oil equivalent) per person. Renewable energy innovation per capita is the total number of patents (all technologies) filled for renewable energy generation per million people. Real GDP per capita is real gross domestic product (in 2017-dollar price) per person based on purchasing power parity or PPP. The producer price index is based on all commodities (2010 = 100). As the producer price index is not available in India, we use the wholesale price index (2010 = 100) for the country and the index is based on the mix of industrial and agricultural products at several stages of production and distribution. The CO_2_ per capita is the total CO_2_ emissions in tonnes per person.

**Figure 1:**
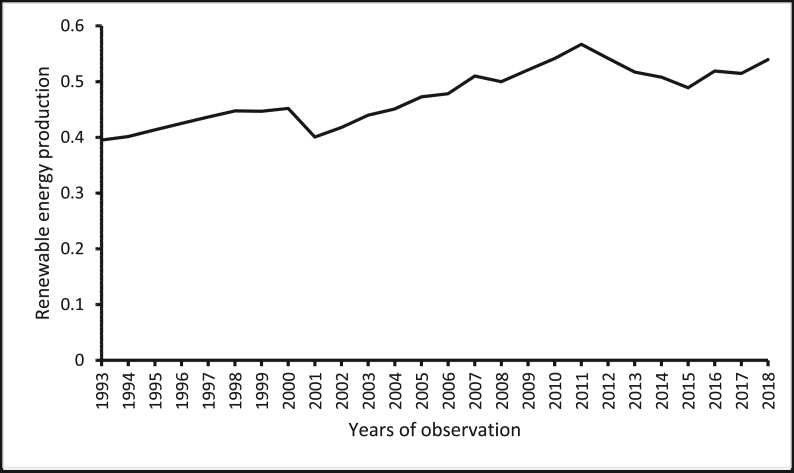
Production of renewable energy (in tonnes) per capita, Brazil.

**Figure 2:**
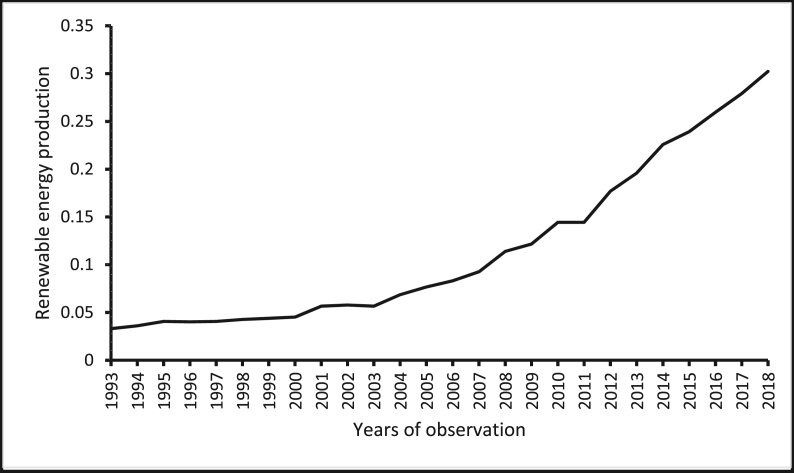
Production of renewable energy (in tonnes) per capita, China.

**Figure 3:**
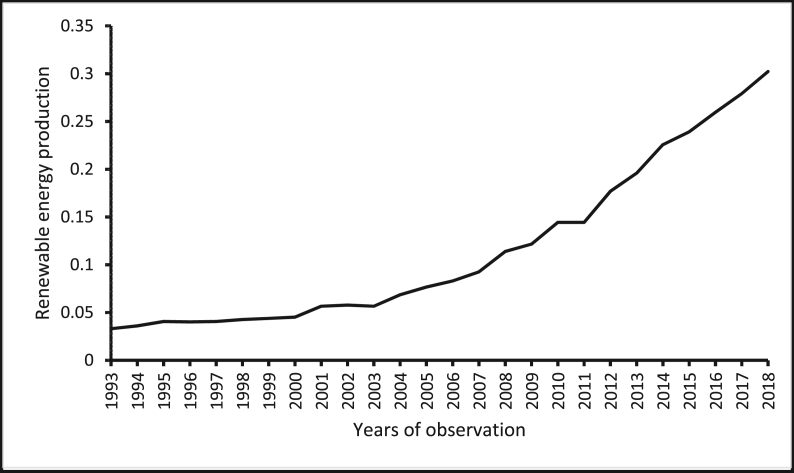
Production of renewable energy (in tonnes) per capita, India.

**Figure 4:**
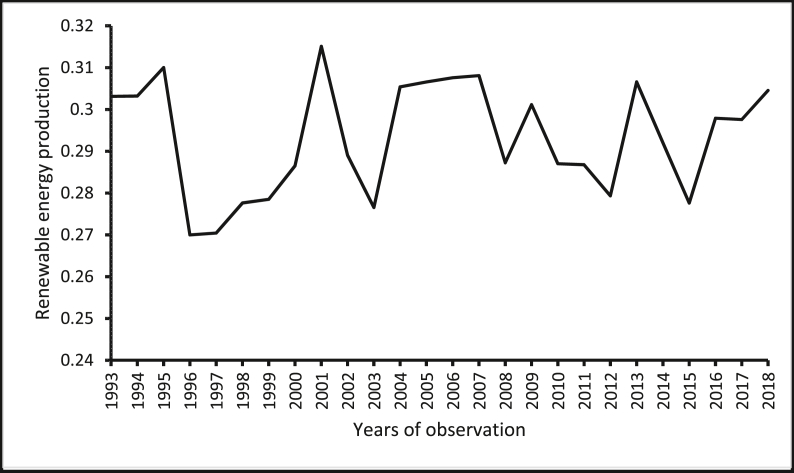
Production of renewable energy (in tonnes) per capita, Russia.

**Figure 5:**
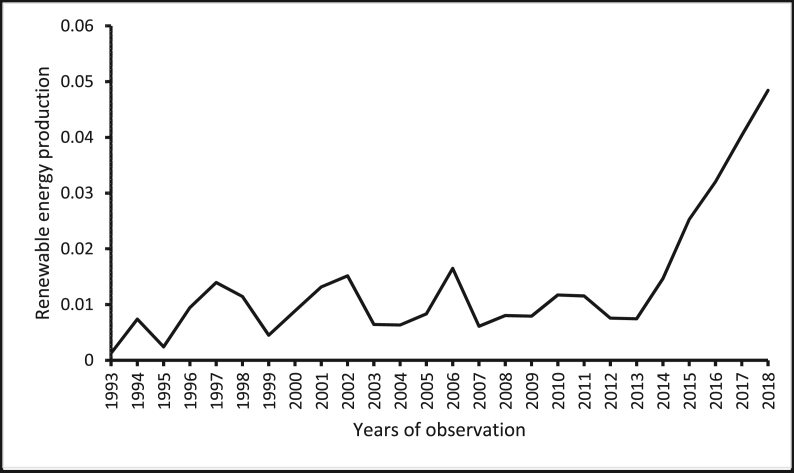
Production of renewable energy (in tonnes) per capita, South Africa.

The source of renewable energy production data is the *Data Section* of the U.S. Energy Information Administration website. The source of renewable energy innovation data is the *Organisation of Economic Co-operation and Development (OECD) Statistics* ((OECD), 2021) of the OECD website. The source of real GDP per capita and population size (which has been used to normalize renewable energy production, renewable energy innovation, and CO_2_ emissions) wholesale price index (for India) is the *World Development Indicators* of the World Bank (The World Bank, 2021). The sources of the producer price index are the *Statistical Yearbook*s of the National Bureau of Statistics of China. The source of the producer price index for Brazil and South Africa are from the websites of the central banks of these countries. Constraint by the amount of data available, constraints, our focus is restricted to cover the period between 1993 and 2018. Table 1 shows the descriptive statistics of the variables, where the average renewable energy innovation per capita in these countries is reported to be 0.185. The average renewable energy innovation per capita in these countries is less than the average renewable energy innovation per capita in the United States, which is 11.080 as provided by the *OECD Statistics* ((OECD), 2021) and the *World Development Indicators* of The World Bank (2021)*.* Furthermore, the renewable energy production per capita is reported in the five economies for the period, 1993–2018 are presented in Figures 1, 2, 3, 4, 5. It is shown that Brazil and Russia are the nations with the biggest renewable energy production per capita, while India and South Africa are the countries with the smallest renewable energy production per capita.

**Table 1:** Descriptive statistics.

Variables	Mean	Median	Standard Deviation	Minimum Value	Maximum Value
REI	0.185	0.080	0.182	0.001	0.567
REP	1.653	0.782	2.341	0.001	12.944
GDP	11005.890	11409.400	6478.61	1907.334	26656.41
PPI	86.638	94.073	42.203	26656.41	197.473
COE	5.372	4.885	3.799	0.748	12.766

The figures are in original forms.

### 3.2 Method of moment quantile regression

Conventional panel data methods including the fully modified ordinary least squares method of Pedroni (2001) and the dynamic ordinary least squares method of Kao and Chiang (2001) can be used to estimate the relationship in Equation (1), especially the impact of renewable energy innovation on its production. However, these methods cannot estimate the impact of renewable energy innovation on its production differently for countries with different levels of production of renewable energy. For the purpose of making appropriate strategies, it is more interesting to know what occurs in extreme scenarios. Quantile regression is suitable to address these questions. It is an extension of the traditional regression methods and can provide a total representation of a conditional distribution. The equation is specified thus:

(2) $$Q_{y_{i}}\left(\delta |x_{t}\right)=\alpha_{\delta }+x_{t}^{T}\beta_{\delta },$$

where $0<\delta <1,Q_{yi}\left(\delta |x_{t}\right)$ implies δth conditional quantile of $y_{t},$ or the dependent series. $x_{t}$ represents the entire regressors, $\alpha_{\delta }$ and $\beta_{\delta }$ are the unobserved effect and parameters at the δth quantile. Within the conventional mean regression approach, the effects of the determinants in the response variable’s conditional mean are assessed, whereas in the quantile regression approach, $\beta_{\delta }$ is the effect of the determinants in the conditional δth quantile of the conditional distribution.

There are environmental economics papers that have used panel quantile regression because they are worried about not providing for the differential impact of the independent variable as a result of differences in levels of the dependent variable (Bui et al., 2021; Ike et al., 2020). Relative to time series data, the benefits of panel data analysis include the use of more observations, less multicollinearity among series, more efficiency, higher degrees of freedom, and larger sample variability (Baltagi and Baltagi, 2008). Therefore, equation (2) is adjusted into a panel quantile regression form as follows:

(3) $$Q_{y_{it}}\left(\delta |\alpha_{i},x_{it}\right)=x_{it}^{\prime }\beta_{\delta }+\alpha_{i}.$$

As specified in equation (3), the existing panel data quantile regression papers are often based on the assumption that individual effect is a mere location shifter, but does not have any impact on the entire distribution. Besides, these methods do not allow for the likelihood of endogeneity. In a bid to circumvent these concerns, Machado and Silva (2019) introduce a novel moments-augmented panel quantile regression approach. Consistent with Machado and Silva (2019), equation (4) is specified as follows:

(4) $$y_{it}=\alpha_{i}+x_{it}^{\prime }\beta +\theta \left(\partial_{i}+N_{it}^{\prime }\vartheta \right)V_{it},$$

where $N_{it}^{\prime }N_{it}$ refers to a known differentiable modification of $x_{it},$ θ(⋅) is a known $\ell^{2}$ function so that $P\left(\theta \left(\partial_{i}+N_{it}^{\prime }\vartheta \right)\right)=1,$ and $V_{it}$ is a random variable that is not observed. Particularly, $V_{it}⊥x_{it},E\left(V\right)=0,$ while E(|V|)=1. We can generate the following from equation (4),

(5) $$Q_{y_{it}}\left(\delta |x_{it}\right)=\alpha_{i}+x_{it}^{\prime }\beta +\theta \left(\partial_{i}+N_{it}^{\prime }\vartheta \right)r\left(\delta \right),$$

where, $r\left(\delta \right)=F_{V}^{-1}r\left(\delta \right),$ and thus P(V<r(δ))=δ in equation (5). Provided θ(⋅) is the identity function and $N_{it}=x_{it},$ it is possible to simplify Equation (4) into:

(6) $$Q_{y_{it}}\left(\delta |x_{it}\right)=\left(\alpha_{i}+\partial_{i}r\left(\delta \right)\right)+x_{it}^{\prime }\beta +x_{it}^{\prime }\vartheta r\left(\delta \right),$$

where, $\alpha_{i}+\partial_{i}r\left(\delta \right)$ is the δth quantile fixed effect for each panel member in equation (6). Contrary to the conventional quantile fixed-effect methods, this procedure permits the time-invariant element to uniquely impact the conditional distribution of $y_{it}$ across the panel members. The marginal impact of the variable $x_{it,k},$ on the δth quantile of $y_{it}$ is $\beta_{k}+r\left(\delta \right)\times \rho \theta \left(\partial_{i}+N_{it}^{\prime }\vartheta \right)/\rho x_{it,k}.$ Similar to the conventional panel quantile fixed-effect methods, a major setback in this approach is the incidental parameters resulting from the numerous fixed effects used (Lancaster, 2002). The results in the inconsistency of the estimates such that cross-sectional units shift closer to infinity, whereas the number of observations of each panel member is fixed (Galvao and Kato, 2016). Machado and Silva (2019) suggested that their sequential estimation method premised on the method of moment quantile model can address this problem.

## 4 Empirical findings

Before estimating the relationship between the variables under consideration in this study, a few initial checks are conducted to ascertain the underlying features of the variables. We initially assess whether the series are cross-sectionally independent. If the series is not cross-sectionally independent, the results generated from the empirical analysis may be biased. Cross-sectional dependence which may emanate from unobserved common factors can significantly reduce efficiency gains of the panel data method over a time series framework if overlooked (Phillips and Sul, 2003). It is, consequently, vital to consider the issue of cross-sectional dependence in order to generate unbiased estimates. In Table 2, we report the Breusch-Pagan LM test of Breusch and Pagan (1980), the Pesaran CD test and Pesaran scaled LM test of Pesaran (2004), and the Bias-corrected LM test of Baltagi et al. (2012) on an equation involving all the five variables in this paper. The results from all of these tests provide overwhelming evidence to support the hypothesis that the series is cross-sectionally independent.

**Table 2:** Outputs of the cross-sectional independence tests.

Dependent variable	Breusch-Pagan LM test	Pesaran scaled LM test	Pesaran CD test	Bias-corrected LM test
REP	61.161∗∗∗ (0.000)	11.440∗∗∗	0.845 (0.398)	11.340∗∗∗ (0.000)

The probability values are presented in the parenthesis. LM is Lagrange Multiplier. CD is cross-sectional dependence. ∗∗∗ imply significance level at 1%.

Therefore, the unit root tests, cointegration tests, and panel estimation methods should provide for cross-sectional dependence in order to abate the chances of getting biased estimates. The next issue is the unit root test and for the purpose of comparison, we initially report the results of the LLC test of Levin et al. (2002), ADF-Fisher Chi-Square test, and PP-Fisher Chi-Square test of Maddala and Wu (1999), Breitung test of Breitung (2001) and the Im et al. test of Im et al. (2003) in Table 3. The empirical findings suggest that there is evidence of unit root in all the series when at level form. However, when the series are expressed in their first differences, there is evidence for stationarity.

**Table 3:** Outputs of the traditional unit root tests.

Panel A: Variables in level form					
Variables	LLC test	ADF-Fisher Chi Square test	PP-Fisher Chi Square test	Breitung test	Im et al. test
REP	2.114 (0.983)	5.195 (0.878)	3.454 (0.969)	1.471 (0.929)	1.764 (0.961)
REI	0.184 (0.573)	9.834 (0.455)	7.142 (0.113)	0.561 (0.713)	0.429 (0.666)
GDP	0.706 (0.760)	9.430 (0.492)	3.182 (0.977)	0.126 (0.550)	1.012 (0.844)
PPI	6.250 (0.999)	15.659 (0.110)	8.701 (0.100)	0.269 (0.606)	-1.115 (0.132)
COE	1.447 (0.174)	8.641 (0.813)	13.517 (0.196)	0.233 (0.592)	-0.882 (0.189)
Panel B: Variables in first differences					
Variables	LLC test	ADF-Fisher Chi Square test	PP-Fisher Chi Square test	Breitung test	Im et al. test
ΔREI	-7.871∗∗∗ (0.000)	69.276∗∗∗ (0.000)	400.684∗∗∗ (0.000)	-6.522∗∗∗ (0.000)	-8.542∗∗∗ (0.000)
ΔREP	-7.752∗∗∗ (0.000)	61.024∗∗∗ (0.000)	70.597∗∗∗ (0.000)	-4.038∗∗∗ (0.000)	-7.184∗∗∗ (0.000)
ΔGDP	-3.921∗∗∗ (0.000)	26.186∗∗∗ (0.000)	31.148∗∗∗ (0.000)	-3.020∗∗∗ (0.001)	-3.013∗∗∗ (0.001)
ΔPPI	49.858∗∗∗ (0.000)	36.618∗∗∗ (0.000)	437.465∗∗∗ (0.000)	-3.009∗∗∗ (0.001)	-4.249∗∗∗ (0.000)
ΔCOE	-3.890∗∗∗ (0.000)	38.125∗∗∗ (0.000)	38.818∗∗∗ (0.000)	-4.327∗∗∗ (0.000)	-4.509∗∗∗ (0.000)

The bandwidth determination is premised on the Newey-West automatic and Bartlett kernel. Schwarz information criterions has been used to select the lag length. ∗∗∗, ∗∗, ∗ imply the significance at 1%, 5% and 10%. The probability values are presented in the parenthesis. LLC is Levine-Lin-Chu. ADF is PP is Phillips-Perron. Augmented Dickey–Fuller.

The foregoing tests do not provide for cross-sectional dependence. In a bid to objectively ascertain the integrating properties of the examined series, while providing for cross-sectional dependence, we have used the cross-sectionally augmented Dickey–Fuller test of Pesaran (2007). The power of the test is not significantly affected when used in small sample analysis. The empirical results suggest that the series are only stationary when expressed in their first differences in Table 4. This suggests that all the series used in the study are integrated of order one.

**Table 4:** Outputs of the cross-sectionally augmented unit root test.

Variables	Level	Variables	First difference
REP	-2.456 (0.363)	ΔREI	-3.701∗∗∗ (0.000)
REI	-1.459 (0.980)	ΔREP	-2.954∗ (0.061)
GDP	-2.362 (0.450)	ΔGDP	-3.191∗∗∗ (0.017)
PPI	-2.499 (0.325)	ΔPPI	-3.655∗∗∗ (0.001)
COE	-1.753 (0.910)	ΔCOE	-3.620∗∗∗ (0.001)

∗∗∗ and ∗ imply significance levels at 1% and 10% . The critical values for the panel are -2.730, -2.860 and -3.100 at 10%, 5% and 1% levels respectively. The estimates are free of heteroscedasticity and autocorrelation. The probability values are presented in the parenthesis.

We have also considered the possibility of a long-run relationship between the series by conducting tests of cointegration and the results are presented in Table 5. Similar to the unit root analysis, we initially used the Pedroni (2004) cointegration test that does not provide for cross-sectional dependence to examine the possibility of a long-run relationship between the series. Pedroni (2004) proposes a set of residual-based cointegration tests that control for heterogeneity. The null hypothesis is such that cointegration does not exist between the series but the results of the four tests suggest that the null hypothesis can be rejected, which implies there is evidence of cointegration. In the same table, we have also reported the results from four cointegration tests that belong to Westerlund (2007), which provide for cross-sectional dependence. Moreover, the cointegration tests use available information more efficiently relative to the majority of the other residual-based cointegration tests (Westerlund, 2007). The null hypothesis is such that cointegration does not exist between the series, but the results of three of the four tests imply there is evidence of cointegration.

**Table 5:** Outputs of cointegration test results.

Panel A: Pedroni residual-based test for cointegration				
Dependent variable	Panel ADF-Statistic	Panel PP-Statistic	Group ADF-Statistic	Group PP-Statistic
REP	-2.634∗∗∗ (0.004)	-7.014∗∗∗ (0.000)	-2.115∗∗ (0.017)	-2.844∗∗ (0.002)
Panel B: Westerlund Bootstrapped error correction based cointegration				
Dependent variable	$G_{\tau }$	$G_{\alpha }$	$P_{\tau }$	$P_{\alpha }$
REP	-2.636∗∗ (0.022)	-7.360 (0.564)	-9.091 ∗∗∗ (0.000)	-14.899 ∗∗∗ (0.000)

∗∗∗ and ∗∗ imply significance at 1% and 5% levels. ADF is Augmented Dickey–Fuller. PP is Phillips-Perron. Gt and Gα are groups mean statistics while Pt and Pα are panel statistics Schwarz information criteria have been used to select the lag length. The probability values are presented in the parenthesis. For the Pedroni test, the bandwidth determination is premised on the Newey-West automatic and Bartlett kernel For the Westerlund [76] test, the number of bootstraps to generate the bootstrapped p values, which are robust is set to 100.

Having ascertained that there is a possibility of a long-run relationship in the variables, we proceed to estimate the long-run relationship between the variables. For comparative purposes, we initially use the fully modified ordinary least squares method of Pedroni (2001) and the dynamic ordinary least squares method of Kao and Chiang (2001) to estimate the relationship among the variables in Table 6. The fully modified ordinary least squares provide for heterogeneous serial correlation. The dynamic ordinary least squares method uses the lead and lagged forms of the series to allow for endogeneity. The results show that increases in renewable energy innovation can propel an increase in renewable energy generation using both methods, but the fully modified ordinary least squares method coefficient is insignificant. The results also show that a surge in real GDP leads to a growth in renewable energy production but the dynamic ordinary least squares method coefficient is insignificant. The impact of the producer price index on renewable energy production produced mixed evidence. The results also show that an increase in CO_2_ emissions leads to less renewable energy production, but the dynamic ordinary least squares method coefficient is insignificant. Despite the benefits of the foregoing two estimators, they still suffer from setbacks such as not allowing for the quantile dimension of the relationship.

**Table 6:** Outputs of panel quantile estimations.

Fully modified ordinary least squares test		Dynamic ordinary least squares test	
Independent variable	Coefficient	Independent variable	Coefficient
REI	0.035 (0.648)	REI	0.505∗∗∗ (0.003)
GDP	0.218∗∗∗ (0.001)	GDP	0.528 (0.465)
PPI	0.213∗∗∗ (0.009)	PPI	-1.692∗∗ (0.015)
COE	-0.196∗∗∗ (0.003)	COE	-0.005 (0.998)

∗∗ and ∗∗∗ imply significance at 5% and 1%. Where applicable, trend and constant are included in the estimation. The bandwidth determination is premised on the Newey-West automatic and Bartlett kernel. The probability values are presented in the parenthesis.

The results of the panel quantile estimation technique of Machado and Silva (2019) are reported in Table 7. The panel quantile regression findings suggest that the effect of renewable energy innovation on renewable energy production is positive and significant in all the quantiles. Moreover, the coefficients are generally bigger at the small quantiles, which suggests that countries with smaller renewable energy production per capita (India and South Africa) have a higher probability to experience a greater impact of renewable energy innovation per capita than countries with bigger renewable energy production per capita (Brazil and Russia). The empirical findings further indicate that the effect of real GDP renewable energy production is significantly positive across all quantiles. Moreover, the coefficients are generally bigger at the small quantiles, which indicates that countries with smaller renewable energy production per capita are more likely to experience a greater impact on real GDP capita than countries with bigger renewable energy production per capita. The results also reveal that the impact of the producer price index on renewable energy production is negative and significant in all the quantiles. The coefficients are not materially different across all the quantiles. Lastly, the results also reveal that the coefficient of the impact of CO_2_ emission on renewable energy production is negative and significant in all the quantiles. Moreover, the coefficients are generally bigger at the small quantiles, which indicates that countries with smaller renewable energy production per capita are more likely to experience a greater impact of the impact of CO_2_ emission per capita than countries with bigger renewable energy production per capita.

**Table 7:** Outputs of panel quantile estimations.

Independent variable	Location	Scale	Quantiles								
			0.1	0.2	0.3	0.4	0.5	0.6	0.7	0.8	0.9
REI	0.352∗∗∗ (0.000)	-0.048 (0.338)	0.432∗∗∗ (0.003)	0.409∗∗∗ (0.001)	0.383∗∗∗ (0.000)	0.362∗∗∗ (0.000)	0.341∗∗∗ (0.000)	0.320∗∗∗ (0.000)	0.315∗∗∗ (0.000)	0.308∗∗∗ (0.000)	0.300∗∗∗ (0.000)
GDP	1.488∗∗∗ (0.000)	-0.175 (0.218)	1.784∗∗∗ (0.000)	1.700∗∗∗ (0.000)	1.605∗∗∗ (0.000)	1.527∗∗∗ (0.000)	1.450∗∗∗ (0.000)	1.371∗∗∗ (0.000)	1.351∗∗∗∗ (0.000)	1.326∗∗∗ (0.000)	1.296∗∗∗ (0.000)
PPI	-0.454∗∗∗ (0.000)	-0.032 (0.698)	-0.400∗ (0.097)	-0.415∗∗ (0.044)	-0.433∗∗∗ (0.000)	-0.447∗∗∗ (0.000)	-0.461∗∗∗ (0.000)	-0.475∗∗∗ (0.000)	-0.479∗∗∗ (0.000)	-0.483∗∗∗ (0.000)	-0.489∗∗∗ (0.000)
COE	-1.451∗∗∗ (0.000)	0.596∗∗∗ (0.000)	-2.456∗∗∗ (0.000)	-2.173∗∗∗ (0.000)	-1.848∗∗∗ (0.000)	-1.586∗∗∗ (0.000)	-1.321∗∗∗ (0.000)	-1.054∗∗∗ (0.000)	-0.986∗∗∗ (0.000)	-0.902∗∗∗ (0.000)	-0.798∗∗∗ (0.000)

∗∗, ∗ imply significance at 1%, 5% and 10%. Constant is included in the estimation The probability values are presented in the parenthesis.

The location parameters suggest that increases in both renewable energy innovation and the real GDP will increase the average renewable energy production in these countries. The location parameters further indicate that increases in both producer price index and CO_2_ emissions will decrease the average renewable energy production in these countries. The scale parameter suggests that an increase in CO_2_ emissions will increase the dispersion of renewable energy production.

In order to conduct a robustness analysis, we have re-estimated the specification in equation (1) using fixed effects panel quantile regression approach also introduced by Machado and Silva (2019). The benefits of using fixed effects panel quantile regression approach are that it is able to control the time-invariant effects and also circumvents the issue of incidental parameters. The results are reported in Table 8 and the signs on the coefficients generated are very similar to the ones obtained in Table 7.

**Table 8:** Outputs of alternative panel quantile estimations.

Independent variable	Quantiles								
	0.1	0.2	0.3	0.4	0.5	0.6	0.7	0.8	0.9
REI	0.118 (0.144)	0.115 (0.210)	0.113 (0.210)	0.110 (0.150)	0.107 (0.100)	0.102∗ (0.070)	0.099∗∗ (0.050)	0.095∗∗ (0.040)	0.090∗∗ (0.030)
GDP	0.593 (0.260)	0.680 (0.440)	0.737 (0.500)	0.822 (0.390)	0.899∗∗ (0.030)	1.005∗∗ (0.033)	1.089∗∗∗ (0.002)	1.201∗ (0.070)	1.331∗ (0.050)
PPI	-0.041 (0.101)	-0.049 (0,188)	-0.054 (0.200)	0.062 (0.170)	-0.069 (0.130)	-0.079∗ (0.060)	-0.087∗ (0.090)	-0.098∗ (0.080)	-0.110∗ (0.070)
COE	-0.680∗∗ (0.020)	-0.038∗∗ (0.011)	-0.108∗∗ (0.040)	-0.211∗ (0.055)	-0.305∗ (0.055)	-0.435∗∗ (0.05)	-0.538∗ (0.050)	0.675∗ (0.050)	-0.833∗ (0.005)

∗∗∗, ∗∗, ∗ imply significance at 1%, 5% and 10%. The probability values are presented in the parenthesis.

## 4 Discussion of the results

The foregoing results reveal that the impact of innovations on renewable energy sources on the generation of renewable energy is significantly positive across all quantiles. The outcomes are in agreement with the conclusions of Danish and Ulucak (2020) that show environmental technologies promote green growth; and Bamati and Raoofi (2020) that show that technological factors promote renewable energy production. The results further suggest the parameter of real GDP is positive and the coefficient of CO_2_ emissions is negative, which is consistent with the findings of Przychodzen and Przychodzen (2020) on 27 post-socialist transition countries.

There are several rationales for the significant and positive impact of renewable energy innovation on renewable energy production. One of the reasons is the rising financing and investment in renewable energy projects in the BRICS, which has translated into the increases in both quality and quantity of innovations in these countries and ultimately increase in productive capacities in these countries including increasing renewable production. Financial institutions in BRICS actively provide loanable funds for renewable energy producers in these countries. There are several specific examples of financing or investment in renewable energy projects in the BRICS countries. For instance, the National Development Bank of Brazil in collaboration with Japanese banks raised 100 million dollars for the purpose of expanding renewable sources in Brazil (Zeng et al., 2017). In South Africa, around three-quarters of the entire renewable energy project funds are generated through bank and institutional loans.

Besides, the Industrial Development Corporation of South Africa provides funds for renewable energy projects through the purchase of community trust shares (Zeng et al., 2017). The government of India invested about US$189 million in Jawaharlal Nehru National Solar Mission in 2011 (Khare et al., 2013). The European Bank for Reconstruction and Development spent 1.197 billion Euros on the clean energy development of Russia, which entails about 25 projects (Kozlova, 2015). There are also specific pieces of evidence to suggest that progress has been made in terms of innovations in the BRICS countries. The global innovation index complied by *World Intellectual Property Organization* shows that the BRICS countries have made tremendous in terms of innovative quality. In 2020, China ranks sixteenth, India ranks twenty-seventh, Russia ranks twenty-eighth, Brazil ranks twenty-ninth and South Africa ranks thirty-eighth in terms of innovative quality in the globe. Among the middle-income economies, China ranks first, India ranks second, Russia ranks third, Brazil ranks fourth and South Africa ranks eighth in 2020 (World Intellectual Property Organization, 2020).

The positive impact of renewable energy innovation on renewable energy production can also be attributed to the abundant renewable energy resource in BRICS countries. For example, Brazil has abundant water resources for the construction of hydropower plants. China has enormous wind resources in its northeastern parts and widespread biomass energy resources in its rural regions. India is a country located in the tropics and endowed with a mean yearly temperature that is compatible with solar energy development. Hence, there are several opportunities for innovative activities in renewable energy sources and innovations to be used for hydropower, solar, and wind generation. Hence, this is likely to lead to more renewable energy production than when there are no abundant renewable energy resources. This is because the lack of abundant renewable energy resources means that the impact of innovations will not be felt much as such innovations will apply to limited renewable energy resources.

## 6 Conclusion

As BRICS countries are emerging economies and whose future growth transitory or sustainable development will heavily depend upon the availability of the renewable energy sources they have or can generate to meet the need for energy consumption, the study examines the impact of technological innovation on renewable energy production. The study account for real gross domestic product, producer price index, and CO_2_ emissions while addressing the research question and uses data by covering the period from 1993 to 2018. The research question in hand has been addressed by using a new panel quantile regression augmented by the method of moments developed by Machado and Silva (2019) which is robust to skewness, heteroskedasticity, and outliers. For the sake of roubsutness, we have also used a fixed effects panel quantile regression approach as an alternative estimator. Also, the study uses innovations in renewable energy generation per capita to serve as the technological factor as compared to the existing studies which have used other proxies to measure technological factors in the models involving renewable energy production including high technology exports as a share of manufactured exports or research and development expenditures.

Overall, the study finds that the effect of renewable energy innovation on renewable energy production is significantly positive across quantiles. Noteworthy to mention that the coefficients are relatively larger at the small quantiles indicating that countries with smaller renewable energy production per capita such as India and South Africa are more likely to experience the greater impact of renewable energy innovation per capita as compared to the countries with bigger renewable energy production per capita such as China, Brazil, and Russia. The study further finds a positive and significant effect on real GDP and a negative and significant impact of the producer price index on renewable energy production across quantiles.

These findings indicate that to promote renewable energy, these economies should not compromise on the real per-capita GDP growth and renewable energy production can be promoted if inflation is being controlled i.e., higher inflation will hamper renewable energy production. Last but not least, the study finds negative and significant coefficients of CO_2_ emissions on renewable energy production across quantiles. These findings indicate that environmental policy-makers in BRICS economies should put efforts into activities that engendered CO_2_ emissions reductions and therefore a blueprint needs to be prepared with a specific aim to increase renewable energy innovation by boosting real gross domestics product, reducing the cost of production and CO_2_ emissions which will, in turn, affect renewable energy production and will lead to the path of sustainable development.

The findings of this study have significant policy insights, especially within the context of BRICS countries. First, the BRICS countries can promote renewable energy without compromising growth in real GDP per capita. Renewable energy production can be also promoted if inflation is being controlled i.e., higher inflation will hamper renewable energy production. In addition, the study finds negative and significant coefficients of CO_2_ emissions on renewable energy production across quantiles. These findings indicate that policy-makers in BRICS economies should put efforts into the reductions of CO_2_ emissions and therefore a blueprint needs to be prepared with a specific aim to increase renewable energy innovation by boosting real gross domestics product, reducing the cost of production and CO_2_ emissions which will, in turn, affect renewable energy production and will lead to the path of sustainable development which is consistent with the aims of COP26.

Because environmental-related technological innovations can promote green growth, it is recommended that BRICS countries should focus their efforts on the development of more environmentally related and renewable-energy enhanced technologies. Empowered with Fourth Industrial Revolution, expansion in investment in energy technologies, especially clean energy technologies, and a greater outlay on research and development in the renewable energy industry should be prioritized by policymakers. CO_2_ emissions will be reduced by devoting more resources to ecologically friendly technology or research and development in the energy sector. Promoting collaboration on environmental technology development will aid in solving the global climate change challenge as well as regional pollution.

Furthermore, as technical innovation in renewable is capital intensive that requires huge financial investments, it is also recommended that BRICS countries should provide the enabling environments and governance paths that will attract other key stakeholders including research institutes to help in the designing of smart energy grids for present and future needs as well as risk experts to assess the risk of such investments and help get the highest return on investment in technological innovations in renewable energy. The consequent increase in renewable energy deployment will be in line with the COP26 agenda.

As China is already taking the lead in terms of advancement in technological innovations in renewable energy generation, it is also recommended that the rest of the BRICS countries should follow China's lead and adapt to increase renewable energy generation. Preferential levies on renewable power projects, for instance, are part of the positive policy measures that have assisted China to become a front-runner in the clean energy sector. South Africa and India, in particular, might change some of their energy laws to provide additional subsidies and tax exemptions to new renewable energy projects. Finally, the legal and regulatory framework has been enhanced. Finally, enhanced legal and regulatory frameworks are proposed for BRICS countries to expand renewable energy output. It will be a benefit for the renewable energy industry if the legal impediments to employing energy-efficient technologies are addressed in the BRICS nations.

It is instructive to note a limitation of this study as it has not factored in the potentially disruptive effect of Russia’s military invasion of Ukraine on the ability of the BRICS countries to play a critical role in boosting global renewable energy output through technological innovation. Though the event is relatively recent, and the BRICS appears to remain united, avoiding outright condemnation of Russia while calling for negotiation to mitigate the crisis, the situation remains largely unpredictable and complicated. It is just recommended that future research efforts in this strand of literature focussing on the BRICS should factor in the potential impacts of the military invasion of Ukraine by Russia into the analysis.

## Declarations

### Author contribution statement

Sakiru Adebola Solarin: Conceived and designed the experiments; Performed the experiments; Analyzed and interpreted the data; Wrote the paper.

Mufutau Opeyemi Bello: Performed the experiments; Contributed reagents, materials, analysis tools or data; Wrote the paper.

Aviral Kumar Tiwari: Analyzed and interpreted the data; Contributed reagents, materials, analysis tools or data; Wrote the paper.

### Funding statement

This research did not receive any specific grant from funding agencies in the public, commercial, or not-for-profit sectors.

### Data availability statement

The data will be available by the authors upon reasonable request.

### Declaration of interest’s statement

The authors declare no conflict of interest.

### Additional information

No additional information is available for this paper.

## Footnotes

1 BRICS s the acronym for five major emerging economies that comprise of Brazil, Russia, India, China, and South Africa.
